# First-line treatment patterns and outcomes in advanced non-small cell lung cancer in Sweden: a population-based real-world study with focus on immunotherapy

**DOI:** 10.2340/1651-226X.2024.20309

**Published:** 2024-04-21

**Authors:** Gunnar Wagenius, Anders Vikström, Anders Berglund, Stina Salomonsson, Goran Bencina, Xiaohan Hu, Diana Chirovsky, Hans Brunnström

**Affiliations:** a Department of Oncology-Pathology, Karolinska Institutet, Stockholm, Sweden; b Theme Cancer, Medical Unit Head and Neck, Lung, and Skin Tumors, Thoracic Oncology Center, Karolinska University Hospital, Stockholm, Sweden; c Department of Respiratory Medicine, Linköping University Hospital, Linköping, Sweden; d Epistat AB, Uppsala, Sweden; e MSD, Centre of Observational Real-World Evidence, Stockholm, Sweden; f MSD, Centre of Observational Real-World Evidence, Madrid, Spain; g Merck & Co., Inc., Centre of Observational Real-World Evidence, Rahway, New Jersey, USA; h Department of Pathology, Department of Clinical Sciences Lund, Lund University, Lund, Sweden; i Department of Genetics, Pathology, and Molecular Diagnostics, Skåne University Hospital, Lund, Sweden

**Keywords:** non-small cell lung cancer, immunotherapy, real-world evidence, overall survival, PDL-1 testing, class of therapy

## Abstract

**Background and purpose:**

The treatment landscape for patients with advanced non-small cell lung cancer (NSCLC) has evolved significantly since the introduction of immunotherapies. We here describe PD-L1 testing rates, treatment patterns, and real-world outcomes for PD-(L)1 inhibitors in Sweden.

**Materials and methods:**

Data were obtained from the Swedish National Lung Cancer Registry for patients with advanced NSCLC and Eastern Cooperative Oncology Group (ECOG) performance status (PS) 0–2 who initiated first-line systemic treatment from 01 April 2017 to 30 June 2020. PD-L1 testing was available in the registry from 01 January 2018. Kaplan-Meier was used for overall survival (OS) by type treatment and histology.

**Results:**

A total of 2,204 patients with pathologically confirmed unresectable stage IIIB/C or IV NSCLC initiated first-line treatment, 1,807 (82%) with nonsquamous (NSQ) and 397 (18%) with SQ. Eighty-six per cent (NSQ) or 85% (SQ) had been tested for PD-L1 expression, a proportion that increased over time. The use of platinum-based therapy as first-line treatment decreased substantially over time while there was an upward trend for PD-(L)1-based therapy. Among patients with PS 0–1 initiating a first-line PD-(L)1 inhibitor monotherapy, the median OS was 18.6 and 13.3 months for NSQ and SQ NSCLC patients, respectively, while for the PD-(L)1 inhibitor and chemotherapy combination regimen, the median OS was 24.0 months for NSQ and not evaluable for SQ patients.

**Interpretation:**

The majority of advanced NSCLCs in Sweden were tested for PD-L1 expression. Real-world OS in patients with PS 0–1 receiving first-line PD-(L)1 inhibitor-based regimens was similar to what has been reported in pivotal clinical trials on PD-(L)1 inhibitors.

## Introduction

Lung cancer is the most common cancer diagnosed globally and the leading cause of cancer death worldwide [1]. In Sweden, it is the fifth most common cancer, but still the leading cause of cancer death [2]. Non-small cell lung cancer (NSCLC) accounts for approximately 85%–90% of all lung cancers and the majority of all NSCLC patients are diagnosed with advanced stage disease (IIIB/C or IV) [3].

Prior to availability of immunotherapies, front-line therapies for advanced NSCLC mainly included chemotherapies and for those with actionable biomarker mutations, tyrosine kinase inhibitors (TKIs). The treatment landscape of advanced NSCLC has changed dramatically since the introduction of immunotherapies. These include immune checkpoint inhibitors that target PD-1 and PD-L1 which are key pathways hijacked by tumors to suppress immune control [4]. The first PD-(L)1 inhibitor monotherapy receiving the European Medicine Agency’s approval as a first-line treatment for advanced NSCLC was pembrolizumab and approved in April 2017 and the first PD-(L)1 inhibitor-based combination regimen was approved in July 2018 (pembrolizumab in combination with pemetrexed and platinum chemotherapy), followed by other PD-(L)1-based regimens from 2019 to 2021. Immunotherapy with anti-PD-(L)1 agents has been shown to improve long-term survival in advanced NSCLC and is now considered a standard treatment either alone or with chemotherapy for patients with metastatic disease [5–9].

Sweden has a tax-financed health care system that provides a uniform system for lung cancer diagnostics and treatment across geographic areas, socioeconomic-, and age groups (e.g. there are no additional costs or need for private insurance for the individual patient for PD-L1 testing or treatment). The Swedish National Lung Cancer Registry (NLCR) is a population-based quality database that contains details on demographics, diagnostic procedures including testing results, and overall survival (OS) for >95% of lung cancer patients, which allows for comprehensive analysis in a real-world setting.

The aim of the present study was to describe PD-L1 testing rates, treatment patterns, and outcomes from the introduction of immunotherapy among locally advanced and metastatic NSCLC patients in a population-based real-world Swedish setting.

## Material and methods

The Swedish NLCR is a Clinical Quality Register, certified by the Swedish Association of Local Authorities and Regions and includes lung cancer patients diagnosed in 2002 and later with a completeness of 95% compared to the National Swedish Cancer Register, to which reporting is mandatory according to the Swedish law. The NLCR includes detailed information on demographic and clinical characteristics such as the date of diagnosis, ECOG performance status (PS), histopathological diagnosis, stage, and biomarker status. Data on PD-L1 testing and results were available in the registry from 01 Jan 2018. In addition, information was also extracted from the Individual Patient Overview (IPO) which is a part of the NLCR. The IPO was created by an interdisciplinary team involving both physicians and nurses from different hospitals in Sweden together with patient representatives. The aim was to create a user-friendly decision support by collecting longitudinal clinically important information for each patient with lung cancer presented in an interactive graphical display, trying to overcome gaps of the Electronic Healthcare Record. The IPO includes detailed information on follow-up of the patient with regard to surgical procedures, radiation therapy, systemic medical treatments including reason for discontinuation, adverse event reporting, and outcomes.

For the present study, patients aged 18 years and older diagnosed with locally advanced or metastatic (stage IIIB-IV) NSCLC disease with a record of first-line treatment after 01 April 2017, which is the date that the first PD-(L)1 inhibitor was recommended to use as first-line therapy for advanced NSCLC (PD-L1 ≥ 50%) in Sweden, to 30 June 2020 were identified in the NLCR and in the IPO. Data cut-off for the present analysis was 30 June 2021 to ensure a theoretical minimum follow-up of 1 year (time from treatment initiation to data cut-off date). Exclusion criteria included patients with an ECOG PS higher than 2 or unknown ECOG PS at index treatment initiation, patients enrolled in clinical trials, and patients planned for chemo-radiation with curative intent. The study population was divided into a nonsquamous (NSQ) and a squamous (SQ) cohort. The study was completed within the guidelines of the Declaration of Helsinki and approved by the national ethical review board (DNR 2020-01547).

### Statistical methods

Biomarker testing and class of therapy were described by histology groups (NSQ, SQ) using descriptive statistics. In a second step, the class of therapy was described by start year of first-line treatment and histology groups. In a subsequent step to understand real-world outcomes with PD-(L)1 inhibitors, the analysis was restricted to only patients with at least one record of PD-(L)1 inhibitor treatment as combination or as monotherapy. Demographic and clinical characteristics and biomarker testing were then summarized by histology groups for the restricted cohort using descriptive statistics. Continuous variables were described by medians and inter-quartile ranges (IQR) and categorical variables were reported as number and percentages. The time to event approach was used to study OS using the Kaplan–Meier methods among the cohorts of patients by histology and class of first line therapy, including PD-(L1) inhibitor monotherapy, PD-(L1) inhibitor combination, and platinum-based chemotherapy combination therapies, and in the subgroup of patients with ECOG PS 0–1; patients with positive epidermal growth factor receptor (EGFR), ALK, or ROS1 test results were excluded to align with the regulatory approved indications. OS was defined from the start date of first-line treatment until date of death, or last date of follow-up (05 May 2021), whichever came first. Median OS, as well as 12 and 24 months OS rates were presented with corresponding 95% confidence intervals (CI). In addition, reasons for discontinuation of PD-(L)1 inhibitors by histology groups and ECOG PS were presented. No statistical tests were applied, and the statistical analyses were performed using R version 4.1.2.

## Results

A total of 6,814 patients (at least 18 years old at diagnosis) were identified with a record of NSCLC diagnosis between 01 April 2017 and 30 Jun 2020 in the Swedish NLCR (Table 1). Of these, 2,773 patients had at least one record of a first-line treatment between 01 Apr 2017 and 30 June 2020. Patients with stage IA–IIIA disease at diagnosis or at the start of first-line treatment (*n* = 77), ECOG PS 3 (*n* = 102) or 4 (*n* = 6) or missing (*n* = 3), patients enrolled into clinical trials (*n* = 99) or with no information on enrolment (*n* = 11), or who were planned for chemo-radiation therapy (*n* = 271) were excluded (Table 1).

**Table 1:** Study population.

Included	Excluded	Description
6.814	4,041	Patients 18 years or older with a record of NSCLC diagnosis between 10 April 2017 and 30 June 2020
		
2,773		Patients with a record of a first-line treatment between 1 April 2017 and 30 June 2020
	77	Stage IA–IIIA at diagnosis or at start of first-line treatment
2,696		
		ECOG PS
	102	ECOG PS 3
	6	ECOG PS 4
	3	ECOG PS missing
2,585		
	99	Enrollment in clinical trials
	11	Missing information of enrollment in clinical trials
2,475		
	271	Planned for chemo-radiation therapy
**2,204**		**Study population**

PS: performance status; NSCLC: non-small cell lung cancer.

**Figure 1:**
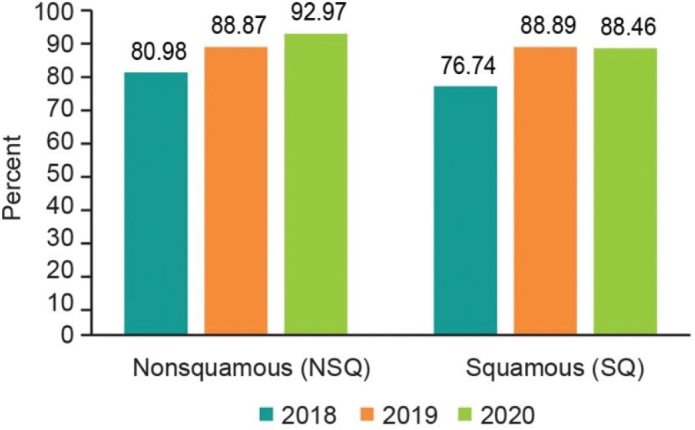
PD-L1 testing by calendar year and by histology.

The final study population thus consisted of 2,204 patients with histologically or cytologically confirmed diagnosis of unresectable stage IIIB/C or IV NSCLC with initiation of first-line treatment between 01 April 2017 and 30 June 2020 and ECOG PS 0–2. The study population was divided into two subgroups: 1,807 (82%) NSQ patients and 397 (18%) SQ patients (Table 2). In 2018 and onwards, from amongst the eligible NSQ patients, 1,256 (99.1%) had a PD-L1 testing status record, of which 1,084 (86.3%) were tested for PD-L1 expression. The corresponding numbers for SQ patients were 276 (98.2%) and 234 (84.8%), respectively. Among the tested NSQ patients, PD-L1 expression was ≥50%, 1%–49%, <1%, and unknown for 402 (37.1%), 317 (29.2%), 310 (28.6%), and 55 (5.1%) patients, respectively. Among tested SQ patients, PD-L1 expression was ≥50%, 1%–49%, <1%, and unknown for 83 (35.5%), 92 (39.3%), 48 (20.5%), and 11 (4.7%) patients, respectively (Table 2). Figure 1 illustrates the PD-L1 testing by calendar year and histology, where there is a clear trend toward an increased testing pattern (Figure 1).

**Table 2:** PD-L1 testing and class of therapy (first-line) by histology.

	Nonsquamous (NSQ)	Squamous (SQ)
	*n* (%)	*n* (%)
**All patients**	1,807 (100.0)	397 (100.0)
All patients between 2018 and 2020	1,268	281
Number of patients with PD-L1 testing status recorded*	1,256 (99.1)	276 (98.2)
Tested for PD-L1*		
Yes	1,084 (86.3)	234 (84.8)
No	172 (13.7)	42 (15.2)
PD-L1 results*		
<1%	310 (28.6)	48 (20.5)
1%–49%	317 (29.2)	92 (39.3)
≥50%	402 (37.1)	83 (35.5)
Unknown	55 (5.1)	11 (4.7)
**Class of therapy, first line**		
Platinum-based chemotherapy combination	889 (49.2)	226 (56.9)
PD-(L)1 inhibitor monotherapy	400 (22.1)	114 (28.7)
PD-(L)1 inhibitor combination	122 (6.8)	21 (5.3)
Anti-VEGF-based combination regimen	55 (3.0)	1 (0.3)
Single agent chemotherapy	77 (4.3)	27 (6.8)
Tyrosine kinase inhibitor therapy	254 (14.1)	6 (1.5)
Non-platinum-based chemotherapy combination	0 (0.0)	1 (0.3)
Other	10 (0.6)	1 (0.3)

* Only available from 2018 and onwards.

**Figure 2:**
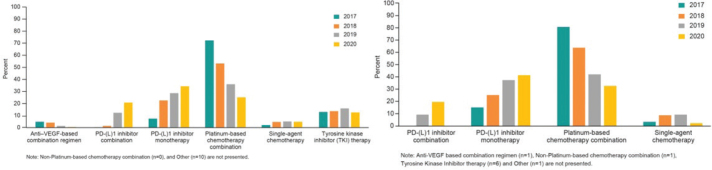
Distribution of class of therapy by calendar year. (A). Distribution of class of therapy by calendar year (first line, nonsquamous). (B) Distribution of class of therapy by calendar year (first line, squamous).

Among NSQ patients receiving first-line treatment, 400 (22.1%), 122 (6.8%), 889 (49.2%), and 254 (14.1%) initiated a PD-(L)1 monotherapy, PD-(L)1 combination therapy, platinum-based chemotherapy, and TKI therapy, respectively. Between 2017 and 2020, the initiation of PD-(L)1 monotherapy increased from 7.4% to 34.3%, and PD-(L)1 combination therapy increased from 0.2% to 20.8% while platinum-based chemotherapy decreased from 72.2% to 25.1%, respectively (Figure 2). The corresponding results for SQ patients were 114 (28.7%), 21 (5.3%), 226 (56.9%), and 6 (1.5%) initiating a PD-(L)1 monotherapy, PD-(L)1 combination therapy, platinum-based chemotherapy, and TKI therapy, respectively (Table 2). The same pattern as for SQ was observed regarding initiated treatment by calendar year, where PD-(L)1 monotherapy increased from 15.1% to 41.3% and PD-(L)1 combination therapy 0%–19.6%, while platinum-based chemotherapy decreased from 80.6% in 2017 to 32.6% in 2020, respectively (Figure 2).

The majority of the NSQ patients initiating first-line PD-(L)1 inhibitor combination were female (55.7%), former smokers (53.3%), had an ECOG PS 1 (62.3%), and stage IV at diagnosis (90.1%). The same pattern was observed among NSQ patients initiating PD-(L)1 inhibitor monotherapy (Table 3). Biomarker testing varied from 65.6% (ROS1) to 92.6% (PD-L1) among NSQ patients initiating PD-(L)1 inhibitor combination, and 67.2% (ROS1) to 94.0% (EGFR) for NSQ patients with PD-(L)1 inhibitor monotherapy (Table 3). Among eligible SQ patients, the demographic and clinical characteristics were similar as for the NSQ patients, except that the majority of patients were male, a larger proportion had stage IIIB–IIIC at diagnosis, and only about half of patients initiating PD-(L)1 inhibitor monotherapy were tested for EGFR/ALK/ROS1 (Table 3).

**Table 3:** Demographic, clinical characteristics, and biomarker status for PD-(L)1 inhibitors at first line by histology.

	Nonsquamous		Squamous	
	PD-(L)1 inhibitor combination	PD-(L)1 inhibitor monotherapy	PD-(L)1 inhibitor combination	PD-(L)1 inhibitor monotherapy
**All patients**	122	400	21	114
Follow-up time*				
Median [IQR]	13.0 [7.4, 18.2]	13.1 [4.6, 22.6]	13.1 [9.4, 17.7]	12.5 [6.0, 19.9]
**Gender**				
Female	68 (55.7)	238 (59.5)	9 (42.9)	44 (38.6)
Male	54 (44.3)	162 (40.5)	12 57.1)	70 (61.4)
**Age at diagnosis**				
Median [IQR]	68.0 [63.0, 72.8]	72.0 [66.0, 76.0]	71.00 [61.00, 74.00]	72.0 [67.0, 78.0]
**Smoking history**				
Current smoker	35 (28.7)	154 (38.5)	6 (28.6)	51 (44.7)
Former smoker	65 (53.3)	209 (52.2)	15 (71.4)	58 (50.9)
Never smoker	22 (18.0)	37 (9.2)	0 (0.0)	5 (4.4)
**ECOG PS**				
PS 0–1	108 (88.5)	320 (80.0)	18 (85.7)	86 (75.4)
PS 2	14 (11.5)	80 (20.0)	3 (14.3)	28 (24.6)
**Stage at diagnosis**				
IIIB	7 (5.7)	34 (8.5)	1 (4.8)	18 (15.8)
IIIC	5 (4.1)	10 (2.5)	5 (23.8)	13 (11.4)
IV	110 (90.1)	356 (89.0)	15 (71.5)	83 (72.8)
**Tested for PD-L1**				
Yes	113 (92.6)	320 (80.0)	20 (95.2)	88 (77.2)
No	6 (4.9)	13 (3.2)	1 (4.8)	5 (4.4)
Unknown	3 (2.5)	67 (16.8)	0 (0.0)	21 (18.4)
**PD-L1 results**				
<1%	31 (27.4)	22 (6.9)	4 (20.0)	5 (5.7)
1%–49%	48 (42.5)	26 (8.1)	13 (65.0)	18 (20.5)
≥50%	25 (22.1)	265 (82.8)	2 (10.0)	64 (72.7)
Undetermined	4 (3.5)	4 (1.2)	0 (0.0)	0 (0.0)
No results reported	5 (4.4)	3 (0.9)	1 (5.0)	1 (1.1)
**Tested for EGFR**				
Yes	111 (91.0)	376 (94.0)	16 (76.2)	62 (54.9)
No	11 (9.0)	24 (6.0)	5 (23.8)	51 (45.1)
Unknown	0 (0.0)	0 (0.0)	0 (0.0)	0 (0.0)
**EGFR results**				
Positive	4 (3.6)	8 (2.1)	1 (6.2)	1 (1.6)
Negative	106 (95.5)	357 (94.9)	14 (87.5)	59 (95.2)
Inconclusive/no response	1 (0.9)	11 (3.0)	1 (6.2)	2 (3.2)
**Tested for ALK**				
Yes	109 (89.3)	363 (90.8)	16 (76.2)	62 (54.4)
No	13 (10.7)	29 (7.2)	5 (23.8)	50 (43.9)
Unknown	0 (0.0)	8 (2.0)	0 (0.0)	2 (1.8)
**ALK results**				
Positive	2 (1.8)	4 (1.1)	0 (0.0)	0 (0.0)
Negative	103 (94.5)	344 (94.8)	15 (93.8)	61 (98.4)
Inconclusive/no response	4 (3.7)	15 (4.1)	1 (6.2)	1 (1.6)
**Tested for ROS-1**				
Yes	80 (65.6)	269 (67.2)	15 (71.4)	51 (44.7)
No	39 (32.0)	64 (16.0)	6 (28.6)	42 (36.8)
Unknown	3 (2.5)	67 (16.8)	0 (0.0)	21 (18.4)
**ROS-1 results**				
Positive	0 (0.0)	3 (1.1)	0 (0.0)	0 (0.0)
Negative	78 (97.5)	258 (95.9)	14 (93.3)	49 (96.1)
Inconclusive/no response	2 (2.4)	8 (3.0)	1 (6.7)	2 (4.0)

IQR: interquartile range; PS: performance status

* Where EGFR/ALK/ROS1 positive patients are excluded.

**Figure 3:**
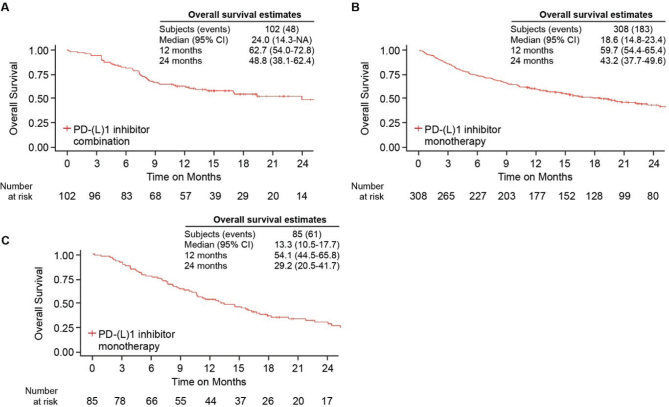
Overall survival in patients initiating a first-line PD-(L)1 inhibitor-based regimen with ECOG performance status 0–1. (A) Overall survival in PD-(L)1 inhibitor combination in patients with nonsquamous non-small cell lung cancer (NSCLC). (B) Overall survival in PD-(L)1 inhibitor monotherapy in nonsquamous NSCLC. (C) Overall survival in PD-(L)1 inhibitor monotherapy in squamous NSCLC.

In patients with ECOG PS 0–1 and without EGFR/ALK/ROS1 mutation, OS estimates by histology and PD-(L1) inhibitors are summarized in Figure 3. The median OS estimates were 24.0 (95% CI 14.3-NA) months and 18.6 (95% CI 14.8–23.4) months for patients with NSQ histology receiving PD-(L)1 inhibitor combination and PD-(L)1 inhibitor monotherapy, respectively. For patients with SQ histology and receiving a PD-(L)1 inhibitor monotherapy, the median OS was 13.3 (95% CI 10.5–17.7) months, while the SQ patients treated with PD-(L)1 inhibitor combination were too few for OS estimation. Further OS data for patients treated with platinum-based chemotherapy, and for the overall cohort of ECOG PS 0–2 patients who received PD-(L)1 inhibitor therapies are found in Supplementary Table 1. The median OS was 9.5 and 11.7 months for platinum-based chemotherapy combination regimens in NSQ and SQ, respectively, in patients with PS 0–1.

Reasons for discontinuation of PD-(L)1 inhibitor by histology groups are summarized in Table 4. The most common reasons for discontinuation in patients who initiated PD-(L)1 inhibitor combination were ‘according to plan’, and ‘disease progression’ in patients initiated on PD-(L)1 inhibitor monotherapy (Table 4).

**Table 4:** Reasons for discontinuation of PD-(L)1 inhibitors by histology.

	Nonsquamous		Squamous	
	Overall	ECOG PS 0–1	Overall	ECOG PS 0–1
**PD-(L)1 inhibitor monotherapy**	400	320	114	86
**Total patients with reason recorded**	331 (82.8)	260 (81.3)	92 (80.7)	72 (83.7)
**Reason for discontinuation**				
According to plan	31 (9.4)	29 (11.2)	9 (9.8)	7 (9.7)
Disease progression	140 (42.3)	113 (43.5)	37 (40.2)	32 (44.4)
Adverse events	58 (17.5)	44 (16.9)	20 (21.7)	15 (20.8)
Patient’s wish	1 (0.3)	0 (0.0)	3 (3.3)	1 (1.4)
Death	35 (10.6)	20 (7.7)	9 (9.8)	5 (6.9)
Other*	66 (19.9)	54 (20.8)	14 (15.2)	12 (16.7)
**PD-(L)1 inhibitor combination**	122	108	21	18
**Total patients with reason recorded**	106 (86.9)	92 (85.2)	13 (61.9)	12 (66.7)
**Reason for discontinuation**				
According to plan	57 (46.7)	52 (48.1)	11 (84.6)	9 (75.0)
Disease progression	13 (10.7)	10 (9.3)	1 (7.7)	1 (8.3)
Adverse events	10 (8.2)	9 (8.3)	2 (15.4)	1 (8.3)
Patient’s wish	1 (0.8)	1 (0.9)	0 (0.0)	0 (0.0)
Death	5 (4.1)	3 (2.8)	1 (7.7)	1 (8.3)
Other*	20 (16.4)	17 (15.7)	0 (0.0)	0 (0.0)

* No additional information available.

## Discussion

The main findings in the present population-based real-world study are that almost all of the patients diagnosed with advanced or metastatic NSCLC were tested for PD-(L)1 expression since 2018. In more recent years, the majority of patients initiated first-line therapy with a PD-(L)1 inhibitor-based regimen, either as monotherapy or in combination with chemotherapy. Nevertheless, platinum-based chemotherapy is still commonly used as the initial first-line treatment, although there’s a decreasing trend over time. In 2020, approximately 25% and 33% of patients with advanced NSQ and SQ NSCLC initiated platinum-based chemotherapy as their first-line treatments. Real-world OS estimates observed in Swedish patients with advanced NSCLC receiving a PD-(L)1 inhibitor-based regimen in first-line therapy were generally in line with findings reported in pivotal PD-(L)1 inhibitor clinical trials as discussed further in the text [6, 8–11].

The treatment landscape in the last years has changed dramatically and emphasizes the need for multidisciplinary teams, which provide better staging, increased adherence to guidelines, and increased survival in patients with NSCLC [12]. After morphological diagnosis, which is crucial to many treatment decisions, the next consideration is treatment-predictive biomarker testing. According to the Swedish National Guidelines for Lung cancer [13] patients diagnosed with advanced or metastatic NSCLC should be tested for PD-(L)1 expression which corroborates with our findings where the majority of the study population had a record of a PD-(L)1 test. Reflex testing for PD-L1 in NSCLC was introduced at all pathology departments in Sweden in late 2016 or early 2017 while reporting to the NLCR did not start until January 2018. In the present study, we could not investigate if a lack of PD-L1 records was due to missing of reporting to the NLCR or if PD-L1 had not been tested due to insufficient tumor material or other reasons. The frequency of PD-L1 positivity seen in our material (≥1% positive tumor cells in approximately 66% of NSQ and 74% of SQ) is in line with or slightly higher than other large real-world studies from Europe and North America showing a prevalence of 52–63% in NSCLC [14–17]. Given their similarity, either of the antibody clones 22C3, 28-8, or SP263 is considered adequate for reflex PD-L1 testing according to Swedish national guidelines, while it is unlikely that any testing with clone SP142 (known to differ in a significant number of cases [18]) has been reported to the NLCR.

Despite the clinical guidelines’ recommendation of using PD-(L)1 inhibitor-based regimens as the standard-of-care first-line treatment options for patients with advanced NSCLC [19, 20], platinum-based chemotherapy was still commonly used as the initial treatment, although there was a decreasing trend over time. Imperfect adherence to guidelines in the time immediately after introduction may be a possible contributor, and also PD-(L)1 combination therapy was not yet regulatory approved for the initial part of the included years, affecting our results. Almost one fourth of the study population had ECOG PS 2 at index date, which might partly explain the usage of platinum-based chemotherapy over immunotherapy in the early years when the clinical experience with PD-(L)1 inhibitors was more limited. Contraindications for immunotherapy may be a further explanation. Unfortunately, we lack data for investigating the reasons for this finding further. It is also noteworthy that part of the patients treated with PD-(L)1 monotherapy exhibited negative or low PD-L1 expression in tumor cells. This may at least partly be explained by adverse effects or contraindications for chemotherapy (and hence its exclusion) in patients planned for PD-(L)1 combination therapy, but further reasons could not be investigated in the present study.

Our results, with real-world OS estimates in patients with advanced NSCLC and ECOG PS 0–1 receiving a PD-(L)1 inhibitor-based regimen in front-line therapy were generally similar to findings reported in pivotal PD-(L)1 inhibitor clinical trials for regulatory approved regimens that were available during the time period of this study. In the 5-year follow-up analysis of KEYNOTE-024, the median OS was 26.3 months (95% CI, 18.3–40.4 months) for pembrolizumab and 13.4 months (9.4–18.3) for platinum-based chemotherapy for NSCLC with PD-L1 expression ≥50% [6]. The 5-year follow-up analysis of the KEYNOTE-189 trial showed a median OS of 19.4 months (15.7–23.4) for pembrolizumab combination therapy and 11.3 months (7.4–16.1) for platinum-based chemotherapy in patients with NSQ NSCLC [21]. Correspondingly, analyses from the IMpower150 and IMPOWER130 trials revealed a median OS for NSQ NSCLC of 18.6–19.5 months for atezolizumab combination therapy approaches and 13.9–14.7 months for platinum-based chemotherapy [8, 22].

In addition, a number of recent European observational studies also assessed OS associated with first-line PD-(L)1 inhibitor-based regimen, with median OS ranging from 12.5 months (95% CI, 9.8–16.4 months) as reported in Dudnik and colleagues to 29.2 months (95% CI, 18.5–39.9 months) as reported by Frost and colleagues [23–29]. The wide range of median OS could be mainly attributable to the differences in study populations, such as distributions of ECOG PS, various duration of follow-up, and inclusion of patients with some unfavorable characteristics (e.g. brain or liver metastases). Real-world survival data associated with PD-(L)1 inhibitor in first-line reported in patients treated in US oncology practice suggested similar implications; in studies where the patient population was restricted to good PS, high PD-L1 expression, and with mature follow-up, median OS was similar to our findings, 19.6 months [30]. Somewhat lower survival rates than in the clinical trials and our analysis were reported in two analyses based on the US Flatiron Health Database, with a median OS of 9.3–12.0 months [31–32]. From the same database, a median OS of 17.2 months was reported for PD-(L)1 combination therapy in NSQ NSCLC [33]. Major differences with the current study include geography, complete information on ECOG PS in the present study, and that our study is population-based, unlike database studies only including patients from a specific US electronic health record system.

The strengths of the present study include the high degree of coverage of patients with NSCLC in Sweden with equal access to health care and advanced diagnostics, detailed information on patient characteristics such as ECOG PS and biomarker testing, and complete death information. The main weakness is the retrospective registry approach with possible errors during entry and missing data. In addition, the follow-up part of the registry, IPO, does not have as high coverage as the NLCR, which could have led to potential selection bias. The overall sample size and the follow-up time were somewhat limited to conduct subgroups analysis and prognostic models. Especially the group of patients with SQ histology receiving PD-(L)1 inhibitor combination therapy was too small for conclusive analysis, and we hope to address this, and also stratification for PD-L1 expression for patients receiving PD-(L)1 inhibitor combination therapy and individual ECOG PS, in the future. Lastly, we were not able to exclude patients who actually received chemo-radiation therapy, but only patients who were planned for it, although this probably did not influence our findings.

## Conclusion

In conclusion, almost all patients diagnosed with advanced NSCLC were tested for PD-L1 expression since 2018 in a Swedish population-based real-world setting. Use of PD-(L)1 inhibitor-based regimens has increased over time representing the majority by 2020, but platinum-based chemotherapy is still commonly used as the initial treatment. Real-world OS estimates observed in Swedish patients with advanced NSCLC receiving a PD-(L)1 inhibitor-based regimen in first line were generally similar to what has been reported in pivotal PD-(L)1 inhibitor clinical trials.

## Supplementary Material

First-line treatment patterns and outcomes in advanced non-small cell lung cancer in Sweden: a population-based real-world study with focus on immunotherapy

## Data Availability

Due to the nature of the research and ethical restrictions the supporting data are not available.
