# Supplementary material for: First-line treatment patterns and outcomes in advanced non-small cell lung cancer in Sweden: a population-based real-world study with focus on immunotherapy
Source: Acta Oncol. 2024 Apr 21;63:20309. doi: 10.2340/1651-226X.2024.20309 (PMC11332554; doi:10.2340/1651-226X.2024.20309)
Supplement: First-line treatment patterns and outcomes in advanced non-small cell lung cancer in Sweden: a population-based real-world study with focus on immunotherapy [file AO-63-20309-s1.pdf]

Supplementary material has been published as submitted. It has not been copyedited or typeset by Acta Oncologica.

Supplementary Table 1. Overall survival (OS) by class of first line therapy, histology, and ECOG PS

	Nonsquamous		Squamous	
**Class of therapy**	**Overall**	**ECOG PS 0-1**	**Overall**	**ECOG PS 0-1**
**PD-(L)1 inhibitor monotherapy**				
Patients	387	308	113	85
Events	246	183	78	61
Median (95% CI)	15.2 (11.9 - 18.6)	18.6 (14.8 - 23.4)	13 (10.5 - 17.7)	13.3 (10.5 - 17.7)
12 months	54.2 (49.5 - 59.4)	59.7 (54.4 - 65.4)	53.9 (45.5 - 64)	54.1 (44.5 - 65.8)
24 months	38 (33.2 - 43.6)	43.2 (37.7 - 49.6)	29.9 (21.9 - 40.8)	29.2 (20.5 - 41.7)
36 months	27.4 (22.1 - 34)	29.9 (23.7 - 37.7)	25 (17.2 - 36.3)	23.2 (14.8 - 36.3)
**PD-(L)1 inhibitor combination**				
Patients	116	102	20	17
Events	60	48	6	4
Median (95% CI)	17.1 (13.1 - NA)	24 (14.3 - NA)	NE	NE
12 months	61.1 (52.9 - 70.7)	62.7 (53.9 - 72.8)	NE	NE
24 months	42.8 (32.8 - 55.7)	48.8 (38.1 - 62.4)	NE	NE
36 months	19.2 (4.6 - 80.1)	22 (5.3 - 91.1)	NE	NE
**Platinum-based chemotherapy combination**				
Patients	841	605	224	161
Events	716	497	190	135
Median (95% CI)	7.8 (7.2 - 8.5)	9.5 (8.5 - 10.5)	9.9 (7.8 - 11.5)	11.7 (10 - 13.9)
12 months	34.5 (31.4 - 37.8)	40.8 (37.1 - 44.9)	42.4 (36.4 - 49.4)	49.7 (42.5 - 58)
24 months	18.9 (16.3 - 21.8)	22.7 (19.5 - 26.4)	24 (18.9 - 30.5)	26.6 (20.4 - 34.7)
36 months	13.1 (10.8 - 15.8)	15.7 (12.9 - 19.2)	12.1 (8.1 - 18.2)	12.7 (8 - 20.1)
